# Supplementary material for: Rapid Self-Assembly of Polymer Nanoparticles for Synergistic Codelivery of Paclitaxel and Lapatinib via Flash NanoPrecipitation
Source: Nanomaterials (Basel). 2020 Mar 20;10(3):561. doi: 10.3390/nano10030561 (PMC7153395; doi:10.3390/nano10030561)
Supplement: Supplementary file 1 [file nanomaterials-10-00561-s001.pdf]

Supplementary Information

Rapid Self-Assembly of Polymer Nanoparticles for Synergistic Codelivery of Paclitaxel and Lapatinib via Flash NanoPrecipitation

Shani L. Levit [1], Hu Yang [1,2,3] and Christina Tang [1,*]

[1] Chemical and Life Science Engineering Department, Virginia Commonwealth University, Richmond, 23284, USA; levitsl@vcu.edu (S.L.L); hyang2@vcu.edu (H.Y.)

[2] Department of Pharmaceutics, Virginia Commonwealth University, Richmond, VA, 23298, USA

[3] Massey Cancer Center, Virginia Commonwealth University, Richmond, VA, 23298, USA

* Correspondence: ctang2@vcu.edu (C.T.)

Received: 17 February 2020; Accepted: 18 March 2020; Published: 20 March 2020

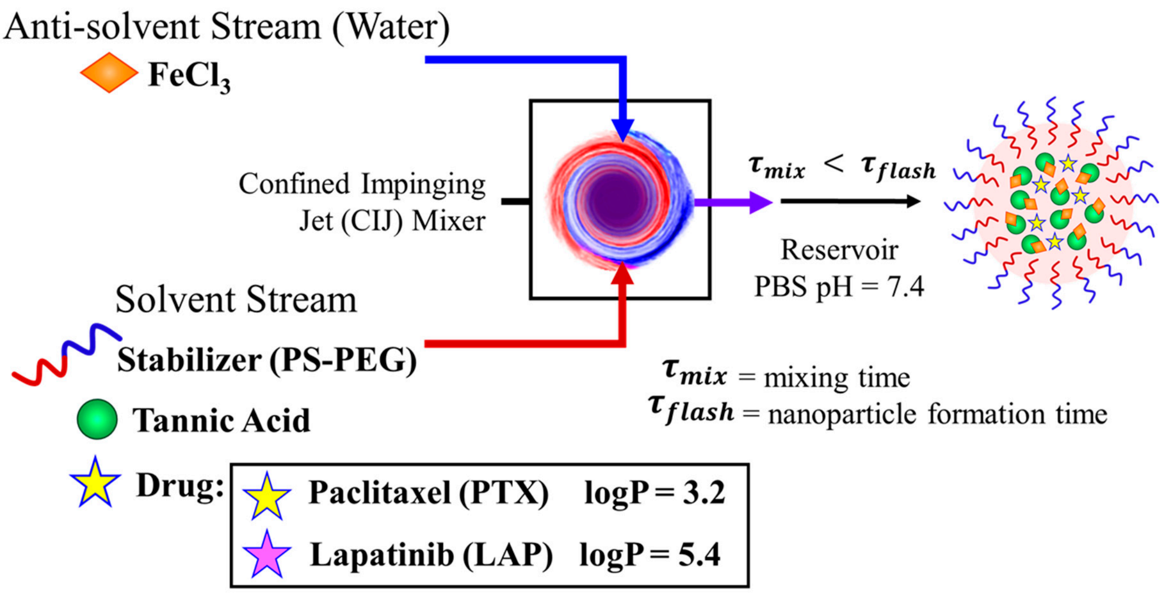

Figure S1. Overview of nanoparticle synthesis with Flash NanoPrecipitation to encapsulate. paclitaxel and lapatinib with a tannic acid (TA) and iron coordination complex using an amphiphilic block copolymer stabilizer. The organic solvent stream contains the stabilizer, PS-b-PEG, TA, and one or more drugs of interest. The organic stream is rapidly mixed with the aqueous stream containing iron using a CIJ mixer. Upon rapid mixing, the TA and iron from an insoluble complex which facilitates the precipitation and encapsulation of paclitaxel and lapatinib. The resulting nanostructures are kinetically trapped.

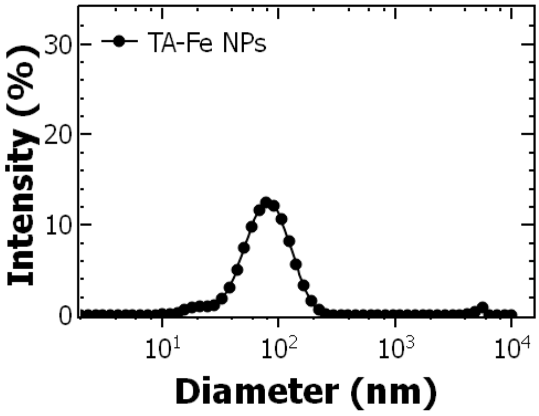

Figure S2. Dynamic light scattering of TA-Fe NPs (containing no drugs).

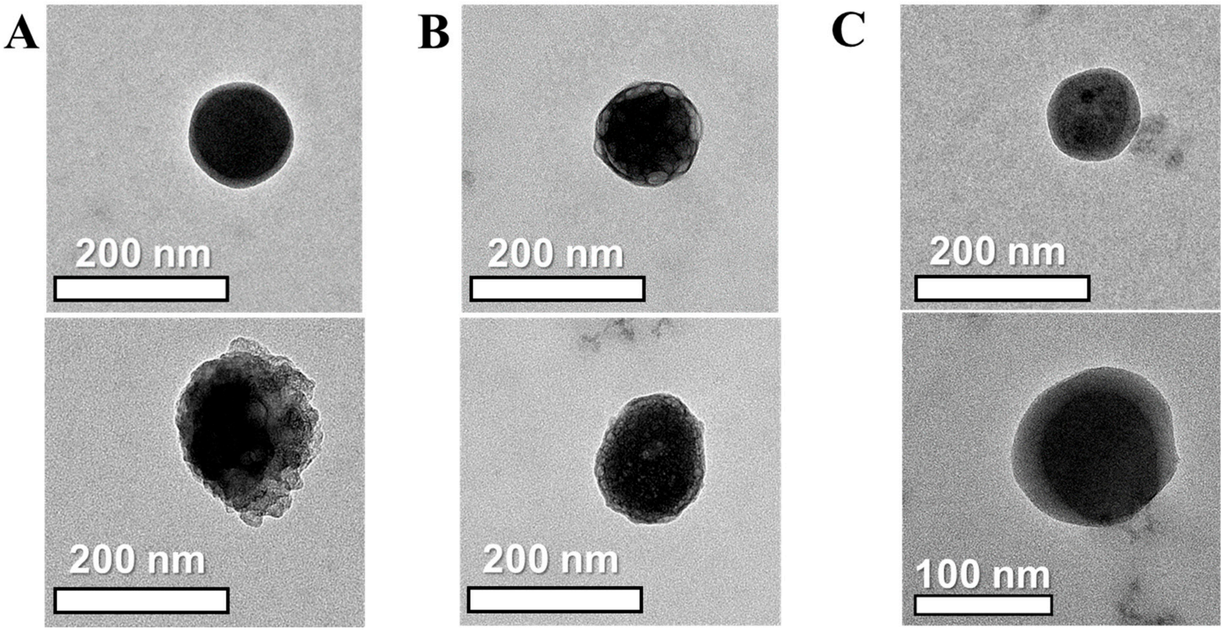

Figure S3. TEM images of (**A**) PTX NPs, (**B**) LAP NPs, and (**C**) PTX-LAP NPs.

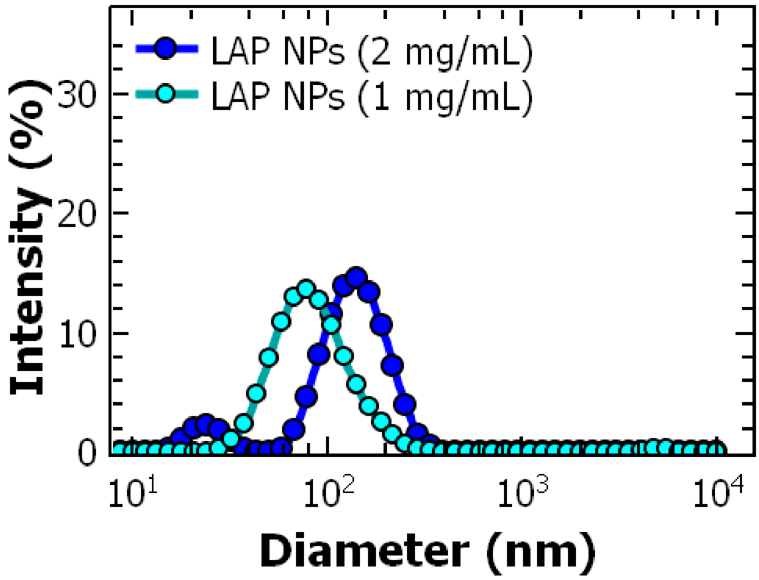

Figure S4. Dynamic light scattering of LAP NPs. Nanoparticles were formulated at 1mg/mL and 2mg/mL drug concentration in the organic stream. The LAP NP dispersion produced at 2 mg/mL had multiple size peaks at ~150 nm and ~30 nm, while the LAP NPs produced at 1 mg/mL were uniform at ~100 nm.

Table S1. Effect of varying drug concentration when preparing for formulating PTX-LAP NPs.

Sample	Drug Concentration (mg/mL)		Size 1 (nm)	Size 2 (nm)	PDI
	PTX	LAP			
PTX-LAP NPs	1	1	119 ± 28	22 ± 3	0.330 ± 0.080
	0.5	0.5	115 ± 3	0	0.248 ± 0.007

Table S2. Comparing nanoparticles size and polydispersity before and after filtration.

Sample	Unfiltered		Filtered		Change in nanoparticle size (%)
	Size (nm)	PDI	Size (nm)	PDI	
TA-Fe NPs	134 ± 3	0.129 ± 0.012	135 ± 5	0.148 ± 0.022	1%
PTX NPs	170 ± 33	0.142 ± 0.053	164 ± 36	0.175 ± 0.050	-4%
LAP NPs	117 ± 7	0.335 ± 0.025	107 ± 8	0.344 ± 0.041	-9%
PTX-LAP NPs	120 ± 35	0.294 ± 0.085	110 ± 12	0.271 ± 0.068	-8%

Table S3. Nanoparticle stability.

Nanoparticles	Initial (T = 0)		T = 2 weeks	
	Size (nm)	PDI	Size (nm)	PDI
TA-Fe NPs	151 ± 5	0.258 ± 0.003	158 ± 2	0.228 ± 0.011
PTX NPs	136 ± 27	0.280 ± 0.059	142 ± 58	0.344 ± 0.225
LAP NPs	117 ± 7	0.247 ± 0.049	132 ± 25	0.332 ± 0.132
PTX-LAP NPs	84 ± 16	0.286 ± 0.026	81 ± 21	0.266 ± 0.058

Table S4. Cell viability of cell treated with Fe-TA NPs.

Total Solids Concentration (µg/mL)	Cell viability
5000	7 ± 1%
1000	38 ± 3%
500	74 ± 5%
100	89 ± 5%
50	95 ± 4%
10	108 ± 4%
5	106 ± 8%
1	101 ± 6%
0.5	100 ± 11%

Table S5. Comparing reproducibility of the IC-50 of OVCA-432 cells treated with free PTX.

Free PTX	IC-50	P-value
trial 1	70.6 ± 5.1	0.379
trial 2	65.8 ± 11.7	

Table S6. Cell viability of cells treated with free PTX and PTX NPs.

Total solids Concentration (µg/mL)	Cell Viability	
	Free PTX	PTX NPs
200	13 ± 3 %	-
150	20 ± 6 %	-
100	26 ± 10 %	-
50	81 ± 24 %	-
20	95 ± 8 %	33 ± 1 %
2	94 ± 7 %	31 ± 2 %
1	-	32 ± 2 %
0.5	-	36 ± 3 %
0.2	88 ± 11 %	39 ± 3 %
0.1	-	43 ± 4 %
0.05	-	63 ± 3 %
0.02	-	88 ± 6 %
0.002	-	98 ± 6 %

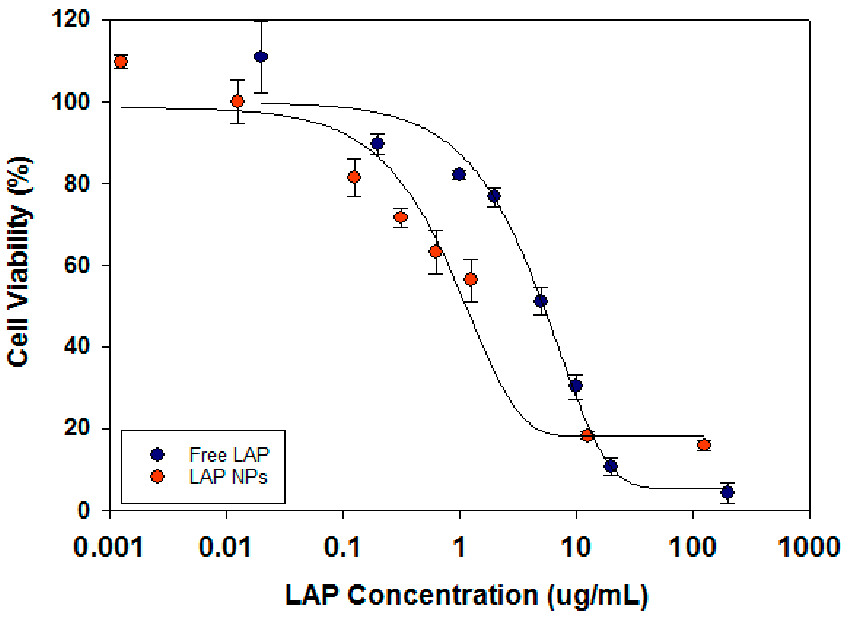

Figure S5. Dose response curve for cell treated with free LAP and LAP NPs.

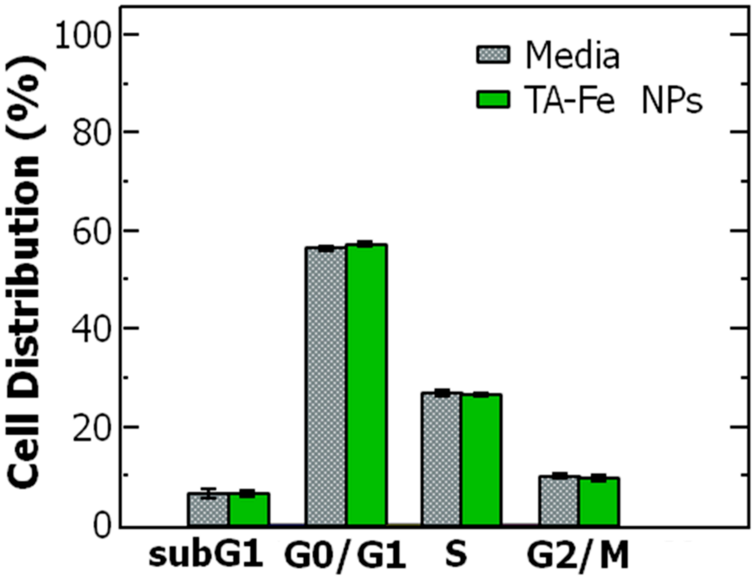

Figure S6. Cell cycle distribution of untreated cells and cell treated with TA-Fe NPs.
